# The situatedness of instructional quality—How situated are college students' ratings?

**DOI:** 10.1111/bjep.70003

**Published:** 2025-07-08

**Authors:** Charlott Rubach, Luise von Keyserlingk, Jutta Heckhausen, Jacquelynne S. Eccles

**Affiliations:** ^1^ Institut für Schulpädagogik Und Bildungsforschung Universität Rostock Rostock Germany; ^2^ Hector Research Institute of Education Sciences and Psychology University of Tübingen Tübingen Germany; ^3^ School of Social Ecology University of California, Irvine Irvine California USA; ^4^ School of Education University of California Irvine California USA

**Keywords:** instructional quality, motivational beliefs, situatedness, state–trait analysis

## Abstract

**Background:**

Student ratings are commonly used to evaluate classroom processes. Research suggests that these ratings are not solely based on objective situational characteristics but also reflect rater characteristics. Although research has been conducted in K‐12 settings, little is known about how person‐specific and situation‐specific factors affect student ratings in higher education.

**Aims:**

This study investigates to what extent students’ ratings on instructional quality (INQ) vary in response to various data collection across teaching situations. In this way, we also investigate the role of motivational beliefs in these ratings.

**Sample:**

The study uses data from 1,745 undergraduates (26.5% self‐identified as male students) from a university in California, U.S.A.

**Methods:**

We employed single trait‐multistate models (STMS) to decompose INQ variance (classroom management, student support, cognitive activation). We estimate the consistency and situation specificity of ratings across courses, time, and course type. Also, we estimated correlations between motivational beliefs and the consistent component across ratings, i.e., the person effect.

**Results:**

High consistency in student ratings of INQ was found within the same course over time. This consistency was lower when INQ was rated across different courses. The consistency was higher in courses that students perceived as difficult than to courses that were perceived as important. Students who reported higher expectancies for success and interest value in these courses also rated INQ more positive across teaching situations.

**Conclusions:**

Our findings suggest that student ratings of INQ are situated and shaped by course characteristics but also rater characteristics. Motivational beliefs contribute uniquely to students’ INQ ratings beyond situational characteristics.

## INTRODUCTION

In educational sciences, student ratings are widely used to capture students' experiences and perceptions of teaching situations over an extended period (e.g., Praetorius, 2014). Prior research has operationalized students' ratings of teaching situations either as stable individual tendencies or as context‐dependent evaluations, thus emphasizing the role of situatedness in student ratings (e.g., Bieg et al., 2022; Gaspard & Lauermann, 2021). Bieg et al. (2022) and Gaspard and Lauermann (2021) focused on student ratings of motivational and affective aspects of teaching, such as teacher enthusiasm. In contrast, Göllner et al. (2018) examined the extent to which student ratings of instructional quality (INQ) are context‐dependent evaluations in K‐12 settings. Although these studies suggest some consistency in student ratings of teaching situations, little is known about how these findings translate to higher education.

This paper extends current research by examining the situatedness of student ratings of INQ in higher education courses. As Rauthmann (2015, *p*.5) stated, ‘each person's rating of a situation contains variance due to the rating person, the rated situation, and the specific person × situation interaction’. An important question to ask in educational science is what information is captured by student ratings of INQ and the extent to how ratings are impacted by rater characteristics, the situation itself and/or represent the person × situation interaction effects (see Göllner et al., 2018).

Trait–state statistical models can help to decompose the variance of student ratings and investigate their situatedness. We use this statistical modelling approach to investigate the consistency and situation specificity in college students' ratings of INQ (rINQ). In particular, we focus on the distinctions between person effects and situation‐specific effects. The *person effect* refers to the consistent inter‐individual tendency to rate INQ across multiple teaching situations (e.g., some students consistently tend to rate cognitive activation generally higher than other students across their classes). The *situation‐specific effects* capture variability in student ratings across different teaching situations. This includes situation effects (e.g., some courses are generally perceived as more supportive by all raters than other courses) and person × situation interaction effects (e.g., a student finds a course highly structured, whereas most classmates rate it as disorganized). We explore whether students' ratings vary across different teaching situations. As a second aim, we investigate the association of motivational beliefs in terms of expectancies for success and interest value (Eccles et al., 1983; Eccles & Wigfield, 2020) with the consistent components of students' ratings of INQ. Our research is conducted in the context of public undergraduate education in California, U.S.A.

## THEORETICAL AND EMPIRICAL BACKGROUND

### Perspectives on instructional quality

It is widely known that INQ plays a major role for students' positive cognitive and affective‐motivational development across educational contexts and stages (Praetorius et al., 2018; Rubach et al., 2022a; Rubach et al., 2022b; Sánchez et al., 2020). INQ is an umbrella concept that refers to teacher behaviour and the interactions between teachers and their students (Decristan et al., 2020; Fauth, Göllner, et al., 2020; Spooren et al., 2013).

INQ is conceptualized as being part of deep structures of instruction. Deep structures are distinguished from surface structures ‐ a distinction that helps to answer the question of how to facilitate learning (Decristan et al., 2020). The surface structure is described by observable strategies and resources used during teaching, that is, media use, social forms, methods and material. Deep structures represent the quality of the interaction between the teacher, students and subject matter. Important in this context is that the orchestration of surface and deep structures with the subject matter influences students' learning and educational success (Klieme, 2022).

Generic frameworks of INQ, which are not specific to a subject and refer to effective practices across subjects, have been suggested by several scholars (see Senden et al., 2022). We make use of the *model of three generic dimensions of instructional quality*,^1^ that is, classroom management, student support and cognitive activation (see Pianta & Hamre, 2009; Praetorius & Gräsel, 2021). The model of three generic dimensions of INQ was developed using a bottom‐up approach, based on data from K‐12 settings. Thus, some scholars define the three dimensions as not theory‐based (Rothland, 2024). The researchers used 21 scales from didactics, teaching research and school climate research for the analysis of classroom videos (Klieme, 2020). These ratings were then examined using factor analyses. It is likely that these generic dimensions also apply to higher education contexts, given the overlap with models of instructional quality in higher education (Helmke & Schrader, 2010; Marsh, 1983; Rubach et al., 2022a; Rubach et al., 2022b). Recent studies have shown that these three dimensions have a (distinct) influence on students' motivational beliefs and achievement (Praetorius et al., 2018; Rubach et al., 2022b; Senden et al., 2022).

*Classroom management* involves effectively organizing activities and time use in class, resulting in, for example, clear rules, structured lessons and minimal disruptions (Evertson & Poole, 2008). *Student support* reflects the extent to which the teacher creates a nurturing and respectful learning environment characterized by emotional and cognitive support, a positive error culture and ensuring choices in class (Praetorius et al., 2018). *Cognitive activation* reflects the extent to which the teacher presents students with challenging tasks, makes connections between different concepts clear and links new content to prior knowledge, thereby enhancing students' understanding, engagement with the content and use of reflection (Leuders & Holzäpfel, 2011; Lipowsky, 2009).

### The situatedness of ratings of instructional quality

**FIGURE 1:**
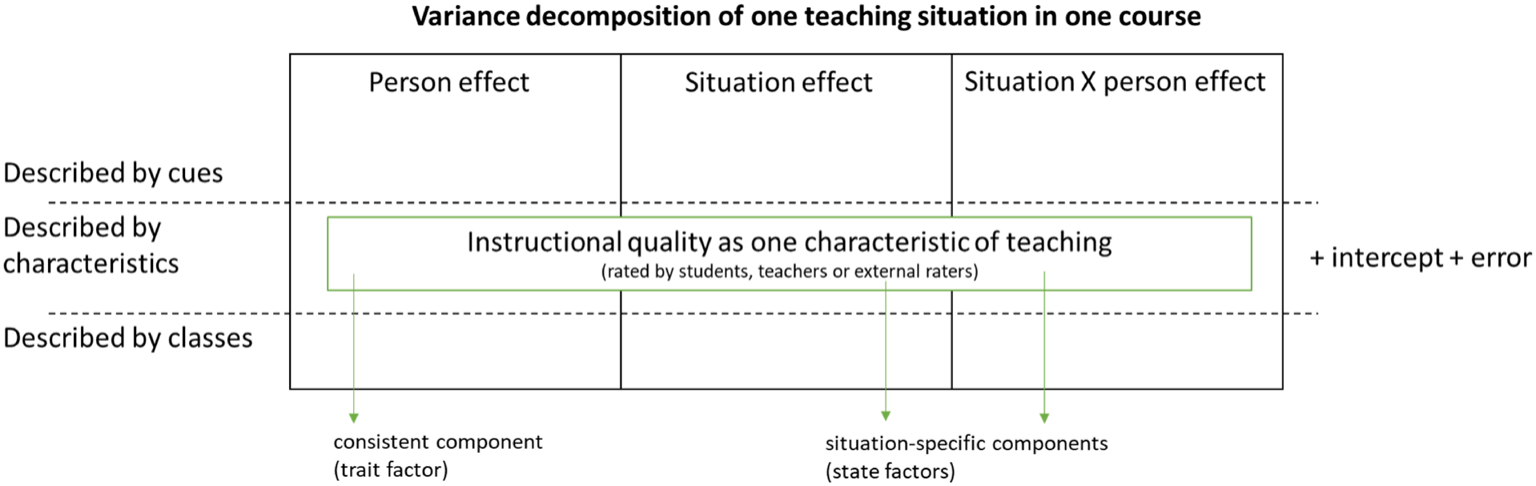
Graphical Representation of the Reality Principle and its corollaries for the example of instructional quality based on Rauthmann et al. (2015).

INQ can be assessed by using multiple data sources (e.g., student ratings, teacher ratings, external rater ratings) and methodologies (e.g., surveys, observations). Student surveys are among the most commonly used methods to measure INQ, that is, students' ratings of their experienced INQ in a particular course (Praetorius, 2014). Some scholars describe students' ratings of INQ as ‘biased’ due to the influence of person‐characteristics and person‐processing of situations (see Beran & Violato, 2005; Spooren et al., 2013), suggesting that bias is problematic. Both Rauthmann et al. (2015) and Lance et al. (2008), along with many psychologists, have argued for a somewhat different perspective. They define intra‐individual and inter‐individual variations in the ratings of situations as meaningful parts of situational experiences and not as bias. In this context, the framework of situations and situatedness helps to conceptualize students' ratings of INQ and their situatedness. There is no universally agreed‐upon definition of ‘situation’ and ‘situatedness’ (see Rauthmann et al., 2015). A working definition by Yang and colleagues (2009, *p*. 1020) suggests that ‘situations can be generally defined as a combination of the individually interpreted, implicit and unique understandings, as well as the culturally shared, explicit and common understandings of the surroundings that produce and constrain human behavior’. Thus, ratings of situations can be decomposed into person effects, situation effects and person × situation interaction effects (+ intercept and error, Rauthmann et al., 2015; see Figure 1). It should be noted, however, that some situations can be so salient that there will be very little person effects, for example, when a person points a gun at you (see Blum et al., 2018). For INQ though, students may share a common perception of INQ. Specifically, students are likely to perceive similar situations differently, or different students may interpret the same situations in varying ways, or the situation may actually differ across students within the same classroom due to differential treatment.

With regard to the situatedness of students' ratings, past studies have examined how their ratings are responsive to various teaching situations that are defined by different times, instructors or subjects (see Gillmore et al., 1978; Marsh, 1983). For example, K‐12 students' ratings show a high level of consistency, that is, a strong person effect, when they evaluate the same instructor in the same course over time (Carpenter et al., 2020; Fauth, Wagner, et al., 2020; Gaertner & Brunner, 2018). Interestingly, Gaertner and Brunner (2018) noted consistency in students' ratings of INQ even when the same K‐12 instructor teaches different subjects. This consistency decreases somewhat when students rate INQ across situations, such as different courses or different instructors at the same or different times (Gaertner & Brunner, 2018; Jaekel et al., 2021; Scherer & Gustafsson, 2015; Wagner et al., 2013). When examining student ratings in higher education, similar patterns of consistencies and variations across different courses and instructors have been found (Daumiller et al., 2023; Feistauer & Richter, 2017). These results show that students' ratings exhibit a moderate degree of consistency, even though they rated INQ in different situations, which suggests a moderate person effect.

Fauth, Wagner, et al. (2020) argued that student ratings of actual instructional behaviour and its consistency vary by INQ dimensions, that is, classroom management, student support and cognitive activation. Some INQ dimensions, such as student support, are more subjective and tied to individual students' needs, whereas others, like classroom management, are perceived more universally (Fauth, Göllner, et al., 2020; Fauth, Wagner, et al., 2020; Göllner et al., 2018; Wagner et al., 2016). Accordingly, K‐12 studies found higher consistency for clarity of instruction, monitoring and classroom management than for students' ratings of emotional or autonomy support (Göllner et al., 2018). These results provide further support for the situatedness of ratings. On the one hand, these findings may reflect situational specificity, that is, the dynamic co‐construction between teachers and students, whereas more individualized instructional processes lead to lower consistency (see Fauth, Göllner, et al., 2020). On the other hand, these results may also indicate a person effect, reflecting students' stable individual needs, attitudes and preferences across learning environments. To our knowledge, only a few studies have systematically examined the situatedness of students' ratings of teaching while also considering different dimensions of INQ (Bieg et al., 2022; Gaspard & Lauermann, 2021; Göllner et al., 2018); no study in higher education has done so.

Another factor that matters for students' ratings of INQ is how difficult students perceive the course (Daumiller et al., 2023; Marsh, 2007; Rubach et al., 2022a; Rubach et al., 2022b). Marsh (1983) argued that the workload and difficulty of the course perceived by students are important background variables for effective teaching. Situated Expectancy‐Value Theory (SEVT, Eccles & Wigfield, 2020), for example, points to the process that perceived difficulty of learning environments can impact how students interpret and behave in these environments due to their motivational beliefs related to their competence and interest. According to Cognitive Load Theory (Plass & Kalyuga, 2019), high cognitive demands might reduce individuals' capacity for detailed information processing, which leads them to rely more on heuristics or general impressions. In such situations, people are more likely to fall back on stereotypes rather than being attentive to situational details (Biernat et al., 2003). Applied to INQ ratings, this suggests that in more cognitively demanding (difficult) courses, students may rely more on general impressions rather than actual situational variations when rating INQ. As a result, their ratings may become more consistent across varying situations, driven more by person effects. Based on SEVT, it can also be assumed that other factors, such as how important students perceive a course to be, can be perceived as situation specificity. In our paper, we focus on the perceived difficulty and perceived importance of courses and how these characteristics determine the situatedness of students' ratings.

### The significance of motivational beliefs for students' ratings of instructional quality

Based on the SEVT (Eccles & Wigfield, 2020) and the theory of social perception (McArthur & Baron, 1983), we hypothesize that students' (initial) motivational beliefs influence their INQ ratings. According to SEVT, the level of students' motivational beliefs, for example, their interest and expectancies for success, influences how they interpret their social environment, for example, their college courses. More specifically, students with stronger motivational beliefs are also more engaged in learning activities, which in turn leads to them receiving greater support from instructors—or perceiving their social reality as more supportive (see Rubach et al., 2020). Marsh (2007) emphasized that the higher the students' interest and expectancies for success, the higher the student's ratings of the teachers' instruction. Other researchers also found that students who are (initially) interested in the course or topic rate the INQ in courses more positively—these links were supported in K‐12 and higher education (e.g., Dahl & Smimou, 2011; Daumiller et al., 2023; Feistauer & Richter, 2018). To our knowledge, only one study did not support the link between initial course interest and students' ratings of teaching (Olivares, 2001). Regarding students' expectancies for success, scholars have found that students with more positive expectations regarding their grades in the course also evaluated the course more positively but not the instructor (e.g., Patrick, 2011). This interpretative lens driven by interest value and expectancies for success may lead to more favorable evaluations across varying instructional settings as students are more consistently (cognitively) engaged across situations (see also Hidi & Renninger, 2006). In contrast, students with weaker motivational beliefs may lack such stable (cognitive) engagement, making them more responsive to situational cues and thus more situated in their rating of INQ (Crawford & Skowronski, 1998; Steinhart & Wyer Jr, 2009).

The theory of social perception suggests that individuals form expectations about their social reality, and these expectations influence information processing, leading to biased perception and judgement formation (McArthur & Baron, 1983)─this perspective sees individual beliefs as the driver of information processing but not their engagement. Nickerson (1998) suggested those expectations influence information processing because either people might only seek information that fit their expectations or because people unconsciously restrict their attention to information that matches their expectations. The impact on ‘biased’ perception and judgement formation depends on the state of motivational beliefs and how much information needs to be processed that matters to the person (see Lord et al., 1979). Crawford and Skowronski (1998), for example, found that people with a high need for cognition were more likely to remember stereotype‐consistent information than people with a lower need for cognition. Thus, highly motivated students might be less flexible in their perceptions of their social reality because the perceived evidence of the social reality matters to them. For INQ ratings in the context of college courses, it can therefore be assumed that students with high interest value and positive expectancies for success might either be (a) more likely to rely on established cognitive schemas or (b) more engaged in courses, resulting in more favorable INQ ratings across different instructional situations.

Even though previous research has provided valuable insights, we still know relatively little about the role of motivational beliefs in shaping different components of student ratings of INQ. In particular, it remains unclear to what extent interest value and expectancies for success influence INQ ratings across multiple situations. We address this research laguna and have two aims: First, to provide insights into the consistency and situation‐specificity of students' ratings of INQ and second to investigate the association between (initial) motivational beliefs and the consistent components of students' INQ ratings. We focus on students' interest value and their expectancies for success.

### The current study

Despite substantial findings providing insights into the situatedness of students' ratings of INQ, several gaps in research remain. First, most existing empirical evidence is derived from K‐12 settings (e.g., Fauth, Göllner, et al., 2020; Fauth, Wagner, et al., 2020; Gaertner & Brunner, 2018; Göllner et al., 2018), whereas studies focusing on higher education are still relatively scarce (e.g., Daumiller et al., 2023; Feistauer & Richter, 2017, 2018). Second, to our knowledge, not many studies have systematically examined the situatedness of student ratings of INQ across various course characteristics and teaching situations. In this study, we examine variations across different courses, time points and types of courses—that is, courses students perceive as their most important or difficult ones. Moreover, the role of motivational beliefs in explaining the consistent component of students' ratings remains an open question (e.g., Göllner et al., 2018). Two research questions are at the centre of our study:

*To what proportion are students' ratings of INQ composed of a consistent component (person effect)* versus *by a situation‐specific component (situation effect, situation* × *person interaction effect)?*

*To what extent are students' motivational beliefs—that is, interest value and expectancies for success —associated with the consistent component of their ratings of the three dimensions of INQ?*

## METHODS

### Sample

Data for this study were collected as part of the UCI‐MUST project (Arum et al., 2021), in which we surveyed multiple cohorts of undergraduates over one academic year at a large public university in California. Within the academic year, these undergraduates participated in weekly surveys in each quarter, that is, the fall, winter and spring quarters. For the current analysis, data from three cohorts from the academic years 2020/21 (*n* = 423), 2021/22 (*n* = 586), to 2022/23 (*n* = 736) were used and combined.

In the full sample (*N* = 1745), 67.6% self‐identified as female students, with 1.7% as other gender identities and 4.3% missing. The ethnic/racial composition included Latiné/Latiné‐American (31.7%), Asian/Asian‐American (38.8%), White/European‐American (12.6%), Black/African‐American (3.7%) and students with other racial/ethnic backgrounds (9.0%; 4.2% missing). Participants' ages ranged from 17 to 65 ($\bar{x}$_age_ = 20, *SD* = 3.00). Furthermore, 50.4% of participants were identified as low‐income (12.1% missing), and 59.7% were first‐generation college‐going students (9.5% missing).

In the UCI‐MUST project, students of all majors were invited to participate in the study. Participating students received course credits. Students who consented to participate were asked in each academic quarter to select one course that they perceived as their most difficult course and one course that they perceived as their most important course in the quarter. Students selected courses as ‘most difficult’ courses, when they were perceived as challenging due to overwhelming material or high workload and low perceived instructional quality. Courses were selected as ‘most important’ when they were perceived as required for students' majors but also courses perceived as personally valuable and important for their future (Rubach et al., 2022a, 2022b).^2^ The selected difficult and important courses spanned a variety of majors, for example, biological sciences, chemistry, mathematics, humanities and psychological science.

### Instruments

All items, results of factor analysis, correlations and reliability indices are represented in the supplementary Tables A, C, D and G: Data S1.

#### Instructional quality (INQ)

**FIGURE 2:**
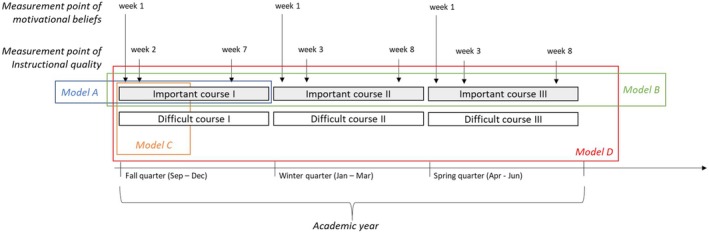
Overview of the design and the timeline of assessment of the UCI‐MUST project. The references to modelling approaches A–D are exemplary and aim to illustrate different analytical strategies. For Models A, B and C, they do not represent an exhaustive list.

In our design, students rated INQ in three self‐perceived difficult and three self‐perceived important courses with one of each type of course in each of three consecutive quarters of one academic year (see Figure 2). Students rated the INQ in their most difficult and most important courses in the second week and the seventh week in the fall quarter and weeks three and eight in the winter and spring quarters (see Figure 2) yielding up to 12 courses being rated per student. As reported by Rubach, von Keyserlingk, et al. (2022b), we adapted existing INQ items (OECD, 2013). Classroom management, student support and cognitive activation were each assessed with three items. The response scale ranged from 1 = *not at all* to 7 = *very much*. Internal consistency was *ω* ≥ .82 in the difficult courses and *ω* ≥ .85 in the important courses.

#### Expectancies for success

Students' initial expectancies for success were assessed with three items at the beginning of the academic quarter for each of the two types of courses (most difficult and most important) each term. Items were developed guided by the expectancy‐value theory (Eccles et al., 2005) and adapted to the college contexts (1 = *not at all good*, 7 = *extremely good*). Factor loadings of the items vary within the scales *λ* ≥ .80 in the difficult courses and *λ* ≥ .79 in the important courses. Internal consistency was *ω* ≥ .87 in the difficult courses and *ω* ≥ .89 in the important courses.

#### Interest value

Students rated their initial interest value beliefs for each type of course at the beginning of each academic quarter. Items were developed guided by the expectancy‐value theory (Eccles et al., 2005) and adapted to the college contexts (1 = *not at all expected*, 7 = *very much expected*). Factor loadings of the items vary within the scales λ ≥ .78 in the difficult courses and λ ≥ .73 in the important courses. Internal consistency (Spearman‐Brown reliability) was r ≥ .76 in the difficult courses and r ≥ .71 in the important courses. The items on expectancies and value belief have already been validated (von Keyserlingk et al., 2022).

### Statistical analysis

To examine the extent to which student ratings of INQ are situated and thus composed of a consistent component and a situation‐specific component, we employ single trait‐multistate statistical models (STMS, see Geiser, 2021). The trait factor in the model captures the consistency (person effect), whereas the state factor in the model represents variations across situations (situation effect, situation × person interaction effect).

To answer RQ1, we estimate the consistency and the occasion‐specificity coefficients (see Geiser, 2021). Occasion specificity is the indicator of situation specificity. The consistency coefficient quantifies the proportion of trait variance in student ratings across multiple situations. The occasion‐specificity coefficient captures the variance attributed to state factors and the situations investigated (Geiser, 2021). Both coefficients represent the proportion of observed [Con (*Y*_it_); Occ (*Y*_it_)] or true (error‐free) score [Con (*τ*_it_); Occ (*τ*_it_)] variability. In this paper, we focus on the true scores.

The complex study design (random‐situational design) with repeated assessments of INQ allowed us to specify four models accounting for multiple situations. These situations are represented by the type of the courses (difficult vs. important course), varying courses (in the fall, winter and spring quarters) and varying time points (two time points per course).

**FIGURE 3:**
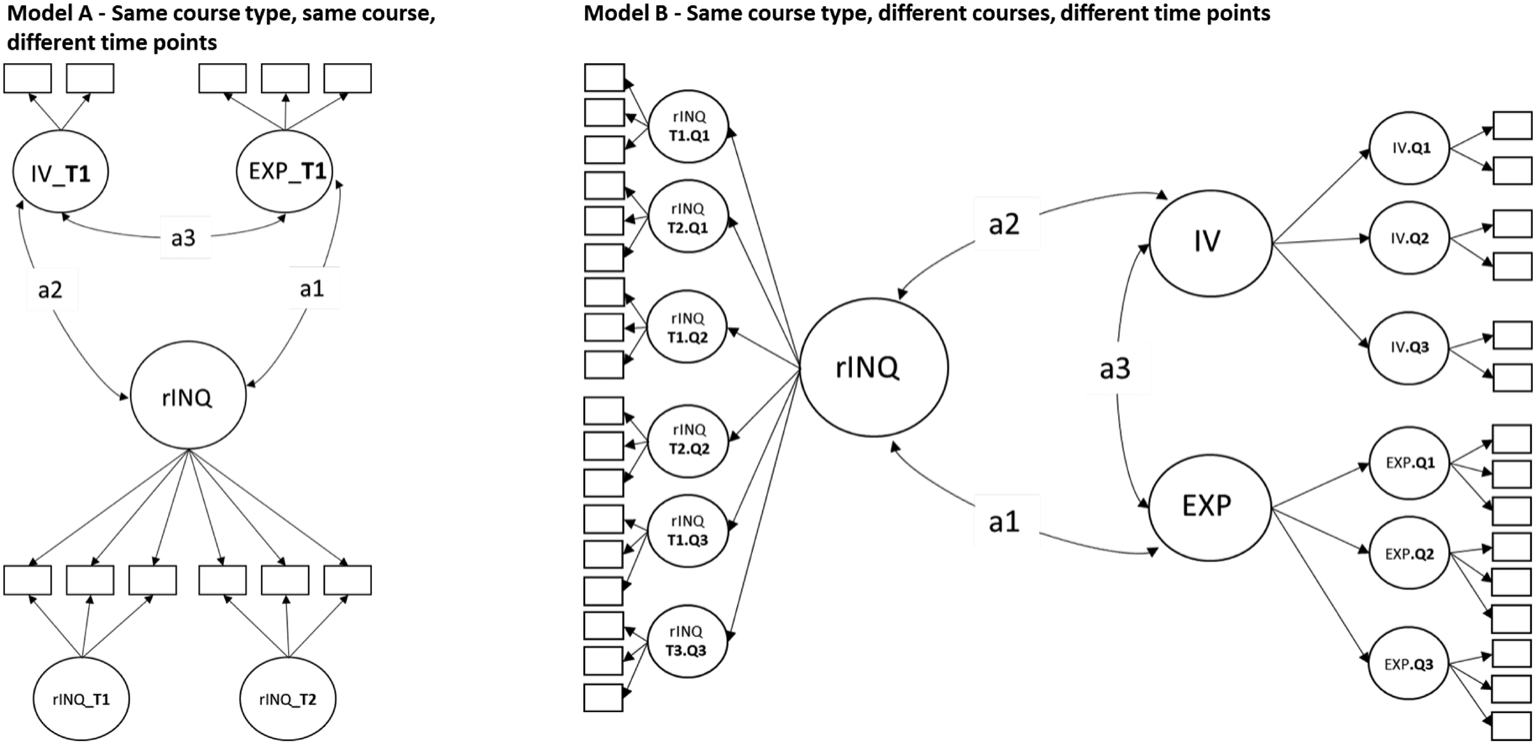
Overview of modelling approaches to Models A and B. EXP, expectancies for success; IV, interest value beliefs; Q1, first quarter of academic year (fall); Q2, second quarter of academic year (winter); Q3, third quarter of academic year (spring); rINQ, rated instructional quality (either classroom management, student support or cognitive activation); T1, time 1 in each quarter; T2, time 2 in each quarter.

Model A ‐ Same course type, same course, different time points: The course type and course are the same, for example, one difficult course, while the time varies. These models enabled us to examine the consistency of each dimension of INQ in one particular course across two time points (see Figures 2 and 3). Here, three models (fall, winter, spring) were specified separately for the difficult and important course in each quality dimension, that is, classroom management, student support and cognitive activation (18 models).

Model B ‐ Same course type, different courses, different time points: Here we specified models of the same course type (difficult or important courses) but three different courses (in fall, winter and spring) and varying time points (six time points; two per quarter). These models allow us to assess the consistency across three courses with the same course perception (important or difficult) and situational variation of each dimension of INQ (occasion specificity, see Figures 2 and 3). Here, one model was specified for each quality dimension for the contexts of the difficult and important courses (6 models).

**FIGURE 4:**
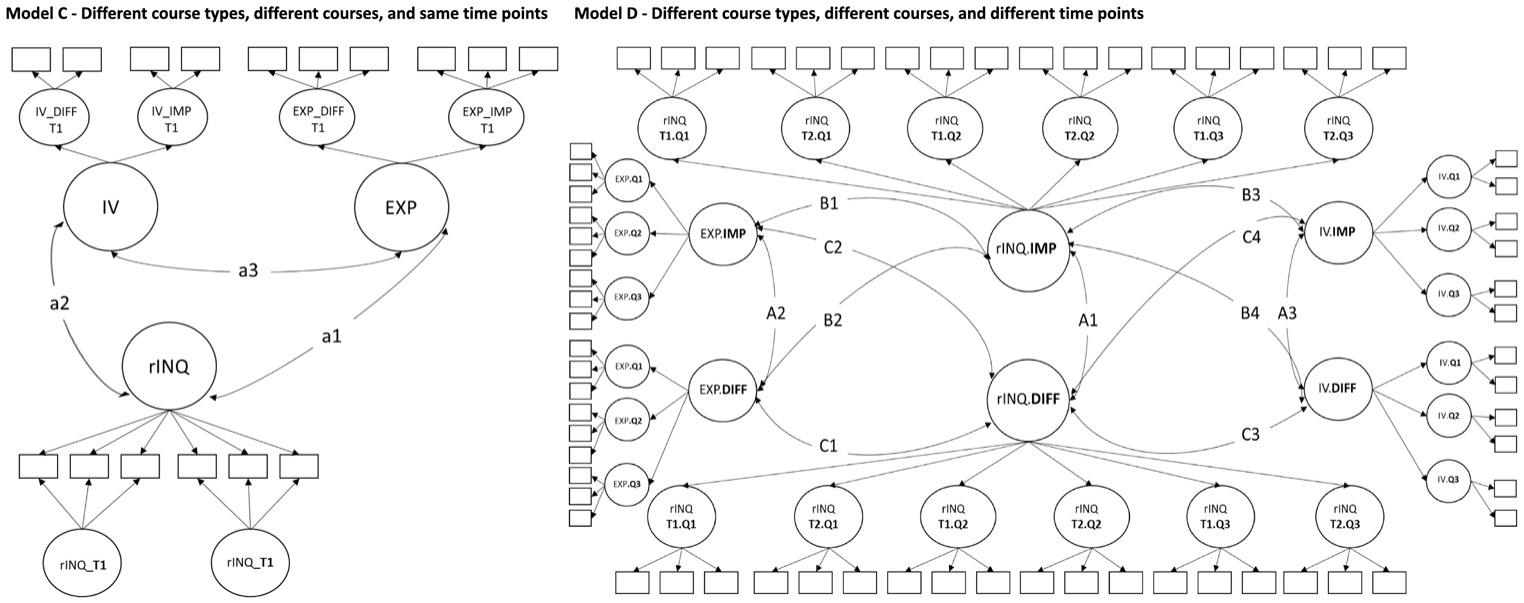
Overview of modelling approaches to Models C and D. DIFF, difficult course; EXP, expectancies for success; IMP, important courses; IV, interest value; Q1, first quarter of academic year (fall); Q2, second quarter of academic year (winter); Q3, third quarter of academic year (spring); rINQ, rated instructional quality (either classroom management, student support or cognitive activation); T1, time 1 in each quarter; T2, time 2 in each quarter.

Model C ‐ Different course types, different courses and same time points: Here we considered the course and course type as variable (important and difficult course), while keeping the time point constant (one time point within the academic quarter). This approach allowed us to investigate the consistency and occasion specificity of each dimension of INQ at a fixed point in time—thus the consistency of ratings across two courses, one important and one difficult, at the same time (see Figures 2 and 4). Here, six models were specified for each quality dimension (18 models).

Model D ‐ Different course types, different courses and different time points: Here the course type (difficult and important courses), the courses and time point (six time points across the academic year; two per academic quarter) were allowed to vary. This approach provided insights into the consistency and occasion‐specificity of each dimension of INQ across time, course and course type—this might be interpreted as the overall perceived INQ at the university (see Figures 2 and 4). One model was specified for each quality dimension (3 models).

To answer RQ2, we added motivational beliefs (*ξ*_EXP_, *ξ*_IV_) into the models and examined the correlations (*ρ*) with the consistent component of INQ (*ξ*_INQ_, person effect). For motivational beliefs, we used different modelling approaches that fit the specifications in Models A–D (see Figures 3 and 4). We also analysed how much of the variance in rating consistency can be explained by motivational beliefs.

For all analyses, we used M*plus* 8.11 (Muthén & Muthén, 1998‐2024) and the robust maximum likelihood estimator (MLR). In all models, we set loadings, intercepts and measurement error variances invariant. By modelling invariant loadings and intercepts, we posit a consistent person effect across situations (see Geiser, 2021). To evaluate the goodness of the models, we compared the less restricted to the more restricted model fits guided by thresholds suggested by Chen (2007) for a sample ≥ 300. All strict measurement invariance models fit the data well (see in the supplementary Tables B1–B3: Data S1).

## RESULTS

### Consistency and occasion specificity of instructional quality (RQ1)

We decomposed the variance in students' ratings of instructional quality (rINQ) into consistency (Con (*τ*_it_)) and occasion specificity (Occ (*τ*_it_)). Results are reported in Table 1. All models showed good model fits (see supplementary Tables E1–E3: Data S1).

**TABLE 1:** Estimates of the true consistency [Con (*τ*_it_)] and occasion‐specificity [Occ (*τ*_it_)] for classroom management, student support and cognitive activation for models with motivational beliefs included.

	Classroom management		Student support		Cognitive activation	
	Con (*τ*_it_)	Occ (*τ*_it_)	Con (*τ*_it_)	Occ (*τ*_it_)	Con (*τ*_it_)	Occ (*τ*_it_)
Model A: Same course type, same course, different time points						
DIFFICULT: FALL^a^	.65	.35	.65	.35	.58	.42
DIFFICULT: WINTER^a^	.64	.36	.68	.32	.65	.35
DIFFICULT: SPRING^a^	.64	.36	.51	.49	.53	.47
IMPORTANT: FALL^a^	.57	.43	.60	.40	.58	.42
IMPORTANT: WINTER^a^	.55	.45	.58	.42	.58	.42
IMPORTANT: SPRING^a^	.47	.53	.52	.48	.46	.54
Model B: Same course type, different courses, different time points						
DIFFICULT: FALL–SPRING^b^	.29	.71	.30	.70	.34	.66
IMPORTANT: FALL–SPRING^b^	.33	.67	.32	.68	.34	.66
Model C: Different course types, different courses, same time point						
FALL‐W2^c^	.35	.65	.30	.70	.33	.67
FALL‐W7^c^	.33	.67	.28	.72	.28	.72
WINTER‐W2^c^	.41	.59	.37	.63	.38	.62
WINTER‐W7^c^	.32	.69	.31	.69	.23	.77
SPRING‐W2^c^	.43	.57	.38	.62	.43	.57
SPRING‐W7^c^	.38	.62	.27	.74	.34	.66
Model D: Different course types, different courses, different time points						
DIFFICULT	.30	.70	.31	.69	.34	.66
IMPORTANT	.35	.65	.34	.66	.35	.65

^a^ Same course (type = complex).

^b^ Different courses (but same context: different or important course).

^c^ Two courses (different course: important and difficult).

*Model A (Same course type, same course, different time points)* showed higher consistency than occasion specificity. Up to 68% of the variance reflected consistent person effect, whereas less than 54% was state residual variance (see Table 1). This suggests that students' ratings of INQ were relatively consistent over time for the same courses and course type (person effect), with consistency outweighing situation specificity across all three dimensions.

*Model B (Same course type, different courses, different time points)* showed lower consistency than occasion specificity. Less than 34% of the variance reflected consistency, while up to 71% reflected state residual variance (see Table 1). This indicates that students' ratings of INQ varied more across different difficult or important courses and time points, suggesting a stronger influence of contextual characteristics.

*Model C (different course types, different courses, same time point)* also revealed lower consistency than occasion specificity. Less than 43% of the variance reflected consistent person effects, whereas up to 77% reflected state residual variance (see Table 1). This can be interpreted as low consistency in INQ ratings across time points (person effect) and more occasion specificity in students' ratings of INQ in all three dimensions across various difficult or important courses at the same time point. Thus, even within a single time point, students' ratings differed considerably depending on the course and course type.

*Model D (different course types, different courses, different time points)* showed a similar pattern: less than 35% of the variance reflected consistent person effects, whereas up to 70% reflected state residual variance (see Table 1). This can be interpreted as low consistency in INQ ratings across time points (person effect) and more situation specificity in students' ratings of INQ in all three dimensions across various difficult or important courses at multiple time points.

In summary, students' ratings of INQ showed greater consistency over time when referring to the same course and course type—for example, a single difficult course rated across multiple time points (Models A). With increasingly varying situations (Models B–C), for example, students' ratings for three difficult perceived courses across time (Models B), the situation specificity of students' ratings increased.

Interestingly, as we integrated ratings of more situations across models, we observed some differences in the consistency and occasion specificity depending on the INQ dimensions, the type of courses and the timing of the ratings. First, comparing consistency and occasion specificity across INQ dimensions, no clear differences occurred in Model A (same course type, same course, different time points). In Model B (same course type, different courses, different time points), ratings of cognitive activation had on average higher consistency than ratings of classroom management and student support. In Model C (different course types, different courses, same time point), ratings of classroom management had on average higher consistency than ratings of student support and cognitive activation. In Model D (different course types, different courses, different time points), ratings of cognitive activation had somewhat higher consistency in the difficult courses than in the important courses.

Second, comparing difficult and important courses in Models A, the consistency of the ratings in all three dimensions tended to be higher in students' difficult courses than in the most important courses. No differences occurred in Models B. Third, model approaches A and C made it possible to compare the consistency and occasion specificity across time points. Models A suggest a slightly lower consistency of ratings from fall to spring, whereas no differences occur in Models C.

### Correlations between motivational beliefs and instructional quality (RQ 2)

In the next step, we were interested in the links between students' expectancies for success (EXP) and interest value (IV) for each course and the consistent component of student ratings of INQ (rINQ). Results are presented in Table 2 (Models A–C) and Table 3 (Model D). The amount of explained variance is represented in Table 4.

**TABLE 2:** Correlational patterns (*ρ*) and standard errors (SE) between rated instructional quality (rINQ) with interest value beliefs (IV) and expectancies for success (EXP) in Models A–C.

	Classroom management			Student support			Cognitive activation		
	A1^a^ rINQ–EXP *ρ* (SE)	A2^a^ rINQ – IV *ρ* (SE)	A3^a^ IV – EXP *ρ* (SE)	A1^a^ rINQ–EXP *ρ* (SE)	A2^a^ rINQ – IV *ρ* (SE)	A3^a^ IV – EXP *ρ* (SE)	A1^a^ rINQ–EXP *ρ* (SE)	A2^a^ rINQ – IV *ρ* (SE)	A3^a^ IV – EXP *ρ* (SE)
Model A: Same course type, same course, different time points									
DIFFICULT: FALL	.38 (.04)	.44 (.04)	.55 (.03)	.46 (.03)	.47 (.04)	.55 (.03)	.47 (.03)	.58 (.04)	.55 (.03)
DIFFICULT: WINTER	.29 (.04)	.48 (.04)	.59 (.03)	.35 (.03)	.48 (.04)	.58 (.03)	.35 (.04)	.57 (.03)	.58 (.03)
DIFFICULT: SPRING	.43 (.04)	.58 (.04)	.61 (.03)	.45 (.04)	.59 (.04)	.61 (.03)	.49 (.03)	.65 (.03)	.61 (.03)
IMPORTANT: FALL	.39 (.03)	.46 (.04)	.55 (.03)	.40 (.03)	.43 (.05)	.55 (.03)	.38 (.03)	.52 (.04)	.55 (.03)
IMPORTANT: WINTER	.36 (.04)	.47 (.04)	.52 (.03)	.35 (.04)	.49 (.04)	.52 (.03)	.34 (.04)	.56 (.04)	.52 (.03)
IMPORTANT: SPRING	.39 (.05)	.48 (.05)	.58 (.04)	.36 (.05)	.45 (.05)	.58 (.04)	.43 (.05)	.61 (.05)	.58 (.04)
Model B: Same course type, different courses, different time points									
DIFFICULT: FALL–SPRING	.58 (.04)	.74 (.04)	.82 (.03)	.65 (.04)	.75 (.03)	.69 (.04)	.63 (.04)	.80 (.03)	.54 (.04)
IMPORTANT: FALL–SPRING	.60 (.03)	.68 (.04)	.73 (.03)	.62 (.04)	.71 (.04)	.81 (.03)	.62 (.03)	.80 (.03)	.81 (.03)
Model C: Different course types, different courses, same time points									
FALL‐W2	.62 (.06)	.68 (.06)	.55 (.05)	.75 (.06)	.77 (.07)	.56 (.05)	.65 (.06)	.84 (.07)	.55 (.05)
FALL‐W7	.58 (.06)	.73 (.06)	.55 (.05)	.70 (.062)	.74 (.07)	.55 (.05)	.67 (.06)	.86 (.08)	.56 (.05)
WINTER‐W2	.52 (.05)	.73 (.05)	.48 (.05)	.59 (.06)	.78 (.06)	.49 (.05)	.60 (.06)	.89 (.05)	.49 (.05)
WINTER‐W7	.49 (.06)	.71 (.07)	.48 (.05)	.28 (.05)	.56 (.07)	.30 (.05)	.52 (.08)	.91 (.08)	.49 (.05)
SPRING‐W2	.49 (.05)	.59 (.06)	.59 (.04)	.49 (.06)	.61 (.06)	.59 (.04)	.54 (.05)	.70 (.06)	.59 (.04)
SPRING‐W7	.57 (.06)	.75 (.06)	.59 (.04)	.71 (.07)	.90 (.08)	.59 (.04)	.68 (.06)	.87 (.06)	.59 (.04)

^a^ The tested paths A1, A2, A3 are visualized in Figures 3 and 4; all estimates are significant with *p* ≤ .001.

**TABLE 3:** Correlational patterns (*ρ*) and standard errors (SE) between the consistency of rated instructional quality (rINQ) with interest value beliefs (IV) and expectancies for success (EXP) in Model D.

		Classroom management	Student support	Cognitive activation
		*ρ* (SE)	*ρ* (SE)	*ρ* (SE)
A1	rINQ.IMP with rINQ.DIFF	.83 (.03)	.77 (.03)	.73 (.03)
A2	EXP.IMP with EXP.DIFF	.83 (.04)	.83 (.03)	.86 (.04)
A3	IV.IMP with IV.DIFF	.83 (.03)	.83 (.03)	.86 (.03)
B1	rINQ.IMP with EXP.IMP	.59 (.03)	.61 (.03)	.60 (.03)
B2	rINQ.IMP with EXP.DIFF	.27 (.04)	.33 (.04)	.34 (.04)
B3	rINQ.IMP with IV.IMP	.68 (.04)	.69 (.03)	.79 (.03)
B4	rINQ.IMP with IV.DIFF	.48 (.04)	.52 (.04)	.57 (.04)
C1	rINQ.DIFF with EXP.DIFF	.53 (.04)	.61 (.04)	.63 (.04)
C2	rINQ.DIFF with EXP.IMP	.53 (.04)	.52 (.04)	.51 (.04)
C3	rINQ.DIFF with IV.DIFF	.71 (.04)	.71 (.03)	.80 (.03)
C4	rINQ.DIFF with IV.IMP	.55 (.04)	.52 (.04)	.56 (.04)

*Note*: The paths A1–C4 (first column) are visualized in Figure 4. All estimates are significant with *p* ≤ .05.

Abbreviations: DIFF, difficult course; IMP, important courses; rINQ, perceived instructional quality (either classroom management, student support or cognitive activation).

**TABLE 4:** Explained variances (*R* ^2^) for investigated associations between the consistency of rated instructional quality (rINQ) with interest value beliefs (IV) and expectancies for success (EXP) in Model A–D.

	Classroom management	Student support	Cognitive activation
	*R* ^2^	*R* ^2^	*R* ^2^
Model A: Same course type, same course, different time points			
DIFFICULT: FALL	.22	.28	.37
DIFFICULT: WINTER	.23	.24	.33
DIFFICULT: SPRING	.23	.37	.44
IMPORTANT: FALL	.24	.22	.28
IMPORTANT: WINTER	.24	.25	.32
IMPORTANT: SPRING	.25	.22	.38
Model B: Same course type, different courses, different time points			
DIFFICULT: FALL–SPRING	.55	.59	.69
IMPORTANT: FALL–SPRING	.48	.50	.65
Model C: Different course types, different courses, same time points			
FALL‐W2	.55	.74	.75
FALL‐W7	.57	.66	.79
WINTER‐W2	.57	.67	.82
WINTER‐W7	.54	.56	.83
SPRING‐W2	.38	.40	.52
SPRING‐W7	.59	.87	.81
Model D: Different course types, different courses, different time points			
IMPORTANT	.49	.51	.69
DIFFICULT	.48	.53	.72

In *Model A*, we examined the correlations between students' initial course‐specific motivational beliefs and rINQ across one quarter in either the most important or most difficult courses. For both the difficult and important courses, students with higher initial IV and EXP rated rINQ overall higher (.22 ≤ *ρ* ≤ .65; .22 ≤ *R* ^2^ ≤ .44). In general, IV had a stronger link to rINQ (.43 ≤ *ρ* ≤ .65) than EXP (.22 ≤ *ρ* ≤ .49). Overall, the links were similarly strong in the difficult courses (.22 ≤ *ρ* ≤ .65) and the important courses (.22 ≤ *ρ* ≤ .61) with some indication of stronger correlations between EXP and rINQ in difficult courses (.29 ≤ *ρ* ≤ .49) compared to the important courses (.33 ≤ *ρ* ≤ .43). The explained variances ranged between .22 and .44 for the difficult courses and between .22 and .38 for the important courses. Some differences occurred when comparing the INQ dimensions: The links between EXP and ratings of student support and cognitive activation (.35 ≤ *ρ* ≤ .49) were somewhat stronger than the link of EXP with ratings for classroom management (.29 ≤ *ρ* ≤ .43) in the difficult courses. Also, the links of IV to ratings of cognitive activation (.52 ≤ *ρ* ≤ .65) were stronger than they were to ratings of student support and classroom management in both difficult and important courses (.43 ≤ *ρ* ≤ .59). The explained variance was somewhat higher for cognitive activation (.28 ≤ *R* ^2^ ≤ .44) and student support (.22 ≤ *R* ^2^ ≤ .37) in most models compared to classroom management (.22 ≤ *R* ^2^ ≤ .24).

In *Model B*, we examined the correlations between students' average motivational beliefs across one academic year with their rINQ across the academic year in either the important or difficult courses. For both the difficult and important courses, across the academic year, students with higher IV and EXP rated the INQ items higher (.58 ≤ *ρ* ≤ .80; .48 ≤ *R* ^2^ ≤ .69) than their peers in these same courses. In general, students' IV scores had a stronger link to their rINQ (.68 ≤ *ρ* ≤ .80) than did their EXP (.58 ≤ *ρ* ≤ .63) and these links were somewhat stronger in the difficult courses (.58 ≤ *ρ* ≤ .80) than in the important courses (.61 ≤ *ρ* ≤ .80). The explained variances ranged between .55 and .69 for the difficult courses and between .48 and .65 for the important courses. Some differences occurred comparing the INQ dimensions: The links between both motivational beliefs and students' ratings of student support and cognitive activation were stronger (.62 ≤ *ρ* ≤ .80) than to students' ratings of classroom management (.58 ≤ *ρ* ≤ .74). The explained variance was somewhat higher for cognitive activation (.65 ≤ *R* ^2^ ≤ .69)) and student support (.50 ≤ *R* ^2^ ≤ .59) in every model compared to classroom management (.48 ≤ *R* ^2^ ≤ .55).

In *Model C*, we examined the extent to which the initial motivational beliefs in the important and difficult courses correlated with their rINQ across the important and difficult courses at the same time point (see Figure 4). Overall, students with higher IV and EXP had a higher rating in their rINQ (.28 ≤ *ρ* ≤ .91; .38 ≤ *R* ^2^ ≤ .87). Again, IV had a stronger link to rINQ (.56 ≤ *ρ* ≤ .91) than did EXP (.28 ≤ *ρ* ≤ .75). Some differences occurred comparing the INQ dimensions: The links between EXP and ratings of student support (.28 ≤ *ρ* ≤ .75) were similar strong as the links to ratings of classroom management (.49 ≤ *ρ* ≤ .62) and cognitive activation (.52 ≤ *ρ* ≤ .68). The links between IV and ratings of cognitive activation (.70 ≤ *ρ* ≤ .91) were somewhat stronger than to ratings of classroom management and student support (.56 ≤ *ρ* ≤ .90). Furthermore, rating collected in the fall (.58 ≤ *ρ* ≤ .86) were slightly more strongly correlated with motivational beliefs than those collected in the spring (.49 ≤ *ρ* ≤ .90). The explained variance was somewhat higher for cognitive activation (.52 ≤ *R* ^2^ ≤ .83) and student support (.40 ≤ *R* ^2^ ≤ .87) in every model compared to classroom management (.38 ≤ *R* ^2^ ≤ .59).

In *Model D*, we examined the extent to which the motivational beliefs across the academic year in students' important and difficult courses correlated with rINQ across the academic year in students' important and difficult courses—thus we examined cross‐dimensional links (see Figure 4). Overall, students with higher IV and EXP rated rINQ higher across the academic year (.27 ≤ *ρ* ≤ .80; .48 ≤ *R* ^2^ ≤ .72) than their peers with lower IV and EXP. Again, IV had a stronger link to rINQ (.48 ≤ *ρ* ≤ .80) than EXP (.27 ≤ *ρ* ≤ .63) and correlations were stronger in the difficult courses (.52 ≤ *ρ* ≤ .80) than in the important courses (.27 ≤ *ρ* ≤ .79). The explained variances ranged between .48 and .72 for the difficult courses and between .49 and .69 for the important courses. Some differences occurred when comparing the INQ dimensions: The correlations of IV and EXP were overall stronger for ratings of cognitive activation (.34 ≤ *ρ* ≤ .80) than for ratings of classroom management and student support (.27 ≤ *ρ* ≤ .71). The explained variance was somewhat higher for cognitive activation (.69 ≤ *R* ^2^ ≤ .72) compared to classroom management (.48 ≤ *R* ^2^ ≤ .49) and student support (.51 ≤ *R* ^2^ ≤ .53).

After describing the findings of each model, we now provide a comparative summary of our results: Motivational beliefs and students' ratings of INQ were positively correlated, with stronger associations for IV than for EXP. Furthermore, both IV and EXP were more strongly correlated with the INQ dimension of cognitive activation than classroom management and student support, with differences of up to 21 percent in Model A, up to 17 percent in Model B and up to 30 percent in Model C. In Model D only, we found stronger correlations in the difficult courses than in the important courses; the explained variance differed only by one to three percent. When comparing these models, the correlations were overall the most homogeneous and strongest in Models B, where students rated their motivational beliefs and INQ across the academic year for the same type of course (most difficult or most important). The explained variance, however, was overall the highest in Models C. The most heterogeneous correlations were indicated in Models C and D. Lastly, findings from Models C and D highlighted the relevance of motivational beliefs across different situations for students' ratings of INQ in higher education. This may suggest that IV and EXP function as stable person characteristics influencing how students evaluate instructional quality across situations.

## DISCUSSION

Students' ratings of INQ are assumed to be a composite of person effects, situation effects and person × situation effects. To understand the significance of INQ for college students academic growth, we need to understand which component is captured when asking students about their perception of various INQ dimensions. The present study addressed research gaps in understanding the consistent and situation‐specific components of college student ratings of INQ by taking into account various course characteristics and teaching situations (RQ 1). We used a unique dataset from higher education, where students rated various courses across their first academic year at one university. In the second step (RQ2), we focused particularly on the consistent component in INQ ratings (person effect) and its link to students' (initial) motivational beliefs.

### The consistency and situation specificity hint to the situatedness of students' ratings of INQ

Prior studies identified that students' ratings of INQ vary somewhat across the time of courses, the subject matter, instructor's popularity and/or the perceived difficulty of the course (Fauth, Wagner, et al., 2020; Feistauer & Richter, 2017; Spooren et al., 2013). Related to our first research question, we extended prior research mostly from K‐12 (e.g., Carpenter et al., 2020; Gaertner & Brunner, 2018; Göllner et al., 2018). We found variations in the consistency of ratings across investigated situations, that is, the highest levels of consistency in INQ ratings within the same course with the same instructor across different time points. As expected, the consistency of INQ ratings decreased when taking into account multiple situations, for example, different courses, different time points or both important and difficult courses. We argue that students incorporate their experiences across teaching situations into their ratings, and their evaluations become more nuanced. Nuanced means that their evaluations may reflect more situationally specific experiences when rating INQ across multiple and varied teaching situations. In line with Fauth, Göllner, et al. (2020), the co‐construction between teachers and students within teaching situations matters, and with variations in teaching situations investigated, also the consistency decreases. But even when teaching situations differed, around one third of the variance in students' ratings of INQ can still be attributed to consistent person effects. This suggests that students apply general tendencies in their evaluations, even across varied contexts. As Göllner et al. (2018) concluded, when students evaluate INQ, their general tendency of ratings matters a great deal. Our analyses show that this conclusion is supported in the higher education context too. Thus, for researchers and evaluators to accurately distinguish between the person and the situation‐specific effects, student evaluations in multiple courses have to be examined. By doing so, we might gain a more precise understanding of teaching effectiveness and assess which aspects of students' ratings of INQ contribute most to students' academic growth.

In addition to these results on the consistency in students' ratings of INQ, we also identified differences across characteristics and situations: On average, the consistency of INQ ratings is somewhat higher in difficult courses than in important courses (see Models A). By difficult courses, we refer to those perceived as challenging due to overwhelming material or high workload and lower perceived instructional quality by the students (Rubach, von Keyserlingk, et al., 2022). Based on this definition, one explanation of these results could be that difficult courses elicit stronger emotional and cognitive loads for students, which may limit students' cognitive capacity for detailed information processing across varying contexts, leading students to fall back on general impressions and more consistent ratings (Biernat et al., 2003). This interpretation gains further relevance when considering the findings related to our second research question: motivated students tend to rate INQ more positively in general. How, then, does cognitive load align with high motivational beliefs? Feldon and colleagues (2019) pointed out that more motivated students also have higher loads. Thus, more motivated students might be more ‘biased’ in their perceptions as they remember more stereotype‐consistent information (see Crawford & Skowronski, 1998; McArthur & Baron, 1983). An alternative explanation would be that more motivated students are more constantly engaged in courses and thus more consistent in their INQ ratings (see Hidi & Renninger, 2006). This suggests that consistency is not a consequence of overload and low information processing, but rather of deeper engagement: motivated students—those with stronger interest value and higher expectancies for success—may engage more consistently across teaching situations and thus actively shape their experiences. This engagement, in turn, may reinforce the consistency in their instructional quality ratings, aligning with assumptions of person × environment fit (see Eccles et al., 1993). These processes may result in more consistent INQ ratings for their perceived difficult courses compared to their perceived important ones. Furthermore, reduced openness or lower expectations toward INQ could reflect a form of the Pygmalion effect. Our results support the idea of the Pygmalion effect because we found that students' expectancies of success are more strongly associated with the consistent component of student ratings of INQ in difficult courses than these expectancies are to students' ratings of INQ in their important courses (see Models A and B). Such findings are crucial because they suggest that instructors in difficult courses have to be aware that students' pre‐existing negative expectancies may affect their perceptions of the course, meaning that even when teaching is of high quality, students may not perceive it as such due to their prior experiences. Furthermore, when measuring INQ, it is important to consider how students assess the difficulty of the course.

By taking into account the three dimensions of INQ, we found some differences in the consistency and situation‐specificity across models. Prior studies suggest higher person effects for clarity of instruction, monitoring and classroom management than for emotional or autonomy support, likely because these strategies are more observable and shared among students within and across courses, compared to more relation‐specific aspects such as emotional or autonomy support (Göllner et al., 2018; Wagner et al., 2016). In our study, we extend these findings by investigating the consistency in ratings on the three dimensions of instructional quality, for example, across multiple courses perceived as difficult or important (see Models B) or multiple course types rated across one academic year (see Model D). Our results align somewhat with previous research, showing that student ratings of student support had the highest consistency in the same course (Models A). This result suggests that ratings of student support are particularly shaped by the co‐construction between students and instructors within the same courses and that this relation between students and instructors varies across different courses with different instructors. Ratings of classroom management and, in some instances, cognitive activation, in contrast, were more consistent across multiple courses (see Models B and C). These findings have significant implications for the measurement and modelling of INQ in higher education, namely ensuring the validity of measurement instruments by differentiating between consistent and situation‐specific perceptions and improving the contextual interpretation of student ratings by accounting for the person effects.

### Motivational beliefs are linked to the person effect in student ratings of instructional quality

As Göllner et al. (2018) posit, researchers still know little about which INQ ratings reflect the strongest person effect of student ratings of INQ, and which reflect the strongest indicator of situation‐specific effects. Previous studies using other measures have found that students who are (initially) interested in a course engage more in that course and rate the teaching and teachers in that course more positively than students who are less interested at the start of the course (Feistauer & Richter, 2018; Goos & Salomons, 2017; Griffin, 2004; Marsh, 2007). The same patterns exist for expectancies for success and INQ ratings (Patrick, 2011). Our findings extend these results. In our study, students with higher motivational beliefs (higher expectancies of success, greater interest) also provided more positive ratings on the INQ measures. These findings suggest that the consistent component in student ratings of INQ—person effect—is at least partly explained by student motivational beliefs (see also Griffin, 2004). To illustrate this using the results from Models C: if the constant component of student ratings averages 37% for classroom management and 33% for cognitive activation, and if motivational beliefs explain on average 53% and 75% of the variance in these components, respectively, this suggests that approximately 20% to 25% of the total instructional quality rating can be attributed to motivational beliefs. This insight is particularly relevant for interpreting student evaluations, especially when they are used to draw inferences about instructional quality and teaching effectiveness, for example for motivational beliefs. Our results suggest that motivational beliefs are not merely shaped by students' perceptions of INQ, but that they also significantly influence how students rate INQ. As described above, it might be that higher motivational beliefs lead to more stereotype‐consistent processing of information or that more motivated students are more consistently engaged across courses, which in turn results in more consistent and more positive INQ ratings across situations.

Our results also suggest that course characteristics influence the extent to which motivational beliefs drive their INQ ratings. For cognitive activation, particularly in Models A, we observe not only that more motivated students report more positive INQ ratings, but also that the associations appear to be stronger in difficult courses—suggesting that motivated students provide more positive ratings in challenging learning environments compared to low‐motivated students. As already mentioned, our results might align with assumptions from person × environment fit theory (see Eccles et al., 1993; Rubach et al., 2022a), which would suggest that students who are more interested or have higher expectancies for their success in these courses may experience a stronger match with challenging courses—resulting in more positive instructional quality ratings.

### Limitations and future research

Our study faces several limitations that should be acknowledged. First, Goos and Salomons (2017) highlighted that the use of data from a single university limits the external validity of our findings. However, our findings are quite consistent with prior studies of K‐12 institutions, as well as the very few papers from higher education, suggesting that external validity may not be a major problem. In the future, it would be good to replicate results across multiple institutions and thus use institutions as another situational characteristic.

Second, we investigated students' rated INQ as the sole method to assess instructional quality in higher education. With the goal of understanding the situatedness of INQ ratings, we know about the need for external rating to decompose the situation‐specific effect, in detail into the situation effect and the person × situation effect (see Rauthmann et al., 2015). For student ratings, there is the difficulty that consistent ratings and actual changes in teaching can be confounded. Thus, it is crucial to replicate these findings using course observations or external raters in addition to student ratings. Additionally, a key next step is to focus on within‐ and between‐student effects to distinguish stable individual rating tendencies from shared perceptions of INQ (see Fauth, Göllner, et al., 2020; Fauth, Wagner, et al., 2020).

Third, we focus only on students' experiences in their perceived most difficult and most important courses and we did not investigate the course by subject matter. Thus, we did not focus on the course's content, but more on the subjective perception of the course. In our study and also previous research, results pointed to the importance of this differentiation to understand the motivational development of college students. However, our design pointed to two extremes of course, perceived importance and difficulty. Thus, we cannot draw implications for general higher education courses and overall INQ. In the future, it would be valuable to select a larger number of randomly selected courses so that subject matter and global teaching styles could be studied. However, this would create a major subject burden for the participants. Thus, it would need to be part of an institutional approach to course evaluation in universities.

Lastly, a potential limitation of our study is the variation in time intervals between measurements for the same versus different courses (see Geiser, 2021). Although measurements within the same course occurred at consistent intervals within a semester—in the fall quarter during weeks two and seven, and in other semesters during weeks three and eight (five weeks)—measurements for different courses spanned multiple semesters with varying intervals. This variation in elapsed time could act as a confounding factor in Models B and D, which estimate consistency across different courses. Specifically, longer time intervals may allow for changes in students' motivational beliefs, or contextual factors, which could in turn affect consistency in ratings—not due to differences in instructional quality per se, but due to shifting internal or contextual reference frames. Future research should consider controlling for elapsed time more explicitly—either through study design or by modelling it statistically.

## CONCLUSION

In summary, the assessment of instructional quality is complex, as these ratings are situated, as we see both consistency and situation specificity in ratings. This dual nature makes it challenging to interpret course evaluations and emphasizes the need to differentiate between what students bring into learning situations and what happens within them.

LST models helped to extract the consistent person effect when taking into account various teaching situations, that is, different courses, types of courses and time points (see Models B, C and D). Our study highlighted that INQ ratings depend on course characteristics, that is, the number of courses rated, time of courses, and how important or difficult courses are perceived by students. Notably, the consistent component in INQ ratings was moderately to strongly associated with students' motivational beliefs—specifically, their interest value and expectancies of success. These insights contribute to a deeper understanding of the psychological basis of student ratings of instructional quality within higher education.

## AUTHOR CONTRIBUTIONS

**Charlott Rubach:** Conceptualization; funding acquisition; writing – original draft; methodology; visualization; formal analysis. **Luise von Keyserlingk:** Conceptualization; investigation; writing – review and editing. **Jutta Heckhausen:** Project administration; investigation; writing – review and editing. **Jacquelynne S. Eccles:** Project administration; investigation; funding acquisition; writing – review and editing.

## FUNDING INFORMATION

This work was supported by grants from The Andrew W. Mellon Foundation (1806‐05902) and the UCI Education Research Initiative.

## CONFLICT OF INTEREST STATEMENT

We have no conflict of interest to disclosure.

## Supporting information


Data S1.

## Data Availability

The dataset analysed during the current study is not publicly available due to data protection processes declared in the study IRB. The analysed dataset is available from the corresponding author on reasonable request.

## Footnotes

^1^
Another similar model is the CLASS conceptual framework for classroom interactions (see Pianta & Hamre, 2009). Here, also three dimensions are defined (emotional support; classroom organization; instructional support) which are developed based on a literature review and thus guided by developmental theory and research (Pianta et al., 2011).

^2^
A detailed explanation of how students interpreted ‘most important’ and ‘most difficult’ is provided in Rubach et al. (2022a).
