# Supplementary material for: The situatedness of instructional quality—How situated are college students' ratings?
Source: Br J Educ Psychol. 2025 Jul 8;95(Suppl 1):S316–36. doi: 10.1111/bjep.70003 (PMC12427147; doi:10.1111/bjep.70003)
Supplement: Supplementary file 1 — Data S1. [file BJEP-95-S316-s001.docx]

# Supplemental Material

**Part A.** Results of exploratory factor analysis for instructional quality

**Part B.** Results of measurement invariance

**Part C.** Results of reliability indicators for all measurements

**Part D.** Correlation of instructional quality constructs

**Part E**. Model Fit Indices of STMS Models

**Part F.** Covariances and standard errors of links between INQ and motivational beliefs

**Part G**. Items used in the study

**Part H.** Result of mean level differences in instructional quality between important and difficult courses and correlations with motivational beliefs

## Table A1.

Exploratory factor analysis for instructional quality

|  | Model comparison | | |  |  |  |  |
| --- | --- | --- | --- | --- | --- | --- | --- |
|  | χ² | *df* | *p* | *RMSEA* | *CFI* | *TLI* | *SRMR* |
| *Difficult course: Exploratory factor analysis* |  |  |  |  |  |  |  |
| Single-factor solution | 455.60 | 8 | ≤ .001 | .15 | .90 | .87 | .05 |
| 2-factor solution | 420.72 | 7 | ≤ .001 | .12 | .95 | .91 | .03 |
| 3-factor solution | 31.59 | 6 | ≤ .001 | .04 | .99 | .99 | .01 |
| 4-factor solution^1^ | 12.57 | 5 | .03 | .03 | .99 | .99 | .01 |
| *Difficult course: Confirmatory factor analysis*  3-factor solution | 103.47 | 24 | ≤ .001 | .05 | .99 | .98 | .02 |
| *Important course: Exploratory factor analysis* |  |  |  |  |  |  |  |
| Single-factor solution | 541.63 | 8 | ≤ .001 | .15 | .90 | .86 | .05 |
| 2-factor solution | 372.23 | 7 | ≤ .001 | .12 | .95 | .91 | .03 |
| 3-factor solution | 58.61 | 6 | ≤ .001 | .06 | .99 | .98 | .01 |
| 4-factor solution^1^ | 10.60 | 5 | .06 | .02 | .99 | .99 | .00 |
| *Important course:* Confirmatory factor analysis: 3-factor solution | 95.76 | 24 | ≤ .001 | .04 | .98 | .98 | .02 |

*Notes.* ^1^ In this model. we have double loadings and factor loading smaller < 0.4.

## Table B1.

Measurement invariance of the factor model describing the structure of classroom management

|  | | | χ² | *df* | *p* | *RMSEA* | *ΔRMSEA* | *CFI* | *ΔCFI* | *TLI* | *SRMR* | *ΔSRMR* |
| --- | --- | --- | --- | --- | --- | --- | --- | --- | --- | --- | --- | --- |
| *Difficult courses* | | |  |  |  |  |  |  |  |  |  |  |
| 1 | Configural invariance | | 323.11 | 120 | ≤ .001 | .032 |  | .982 |  | .977 | .025 |  |
| 2 | Weak invariance | | 335.13 | 130 | ≤ .001 | .031 | .001 | .982 | .000 | .978 | .028 | -.003 |
| 3 | Strong invariance | | 364.75 | 140 | ≤ .001 | .031 | .000 | .980 | .002 | .978 | .031 | -.003 |
| 4 | Strict invariance | | 393.57 | 155 | ≤ .001 | .030 | .001 | .979 | .001 | .979 | .031 | .000 |
| *Important courses* | | |  |  |  |  |  |  |  |  |  |  |
| 1 | | Configural invariance | 250.65 | 120 | ≤ .001 | .026 |  | .988 |  | .985 | .018 |  |
| 2 | | Weak invariance | 269.09 | 130 | ≤ .001 | .025 | .001 | .987 | .001 | .985 | .024 | -.006 |
| 3 | | Strong invariance | 297.50 | 140 | ≤ .001 | .026 | -.001 | .985 | .002 | .984 | .026 | -.002 |
| 4 | | Strict invariance | 321.21 | 155 | ≤ .001 | .025 | .001 | .985 | .000 | .985 | .030 | -.004 |

## Table B2.

Measurement invariance of the factor model describing the structure of student support

|  | | | χ² | *df* | *p* | *RMSEA* | *ΔRMSEA* | *CFI* | *ΔCFI* | *TLI* | *SRMR* | *ΔSRMR* |
| --- | --- | --- | --- | --- | --- | --- | --- | --- | --- | --- | --- | --- |
| *Difficult course* | | |  |  |  |  |  |  |  |  |  |  |
| 1 | | Configural invariance | 601.87 | 118 | ≤ .001 | .050 |  | .959 |  | .947 | .027 |  |
| 2 | | Weak invariance | 628.46 | 128 | ≤ .001 | .048 | .002 | .958 | .001 | .950 | .030 | -.003 |
| 3 | | Strong invariance | 673.61 | 138 | ≤ .001 | .048 | .000 | .955 | .003 | .950 | .032 | -.002 |
| 4 | | Strict invariance | 672.70 | 153 | ≤ .001 | .045 | .003 | .956 | -.001 | .956 | .032 | .000 |
| *Important course* | | |  |  |  |  |  |  |  |  |  |  |
| 1 | Configural invariance | | 491.36 | 118 | ≤ .001 | .044 |  | .965 |  | .954 | .027 |  |
| 2 | Weak invariance | | 504.31 | 128 | ≤ .001 | .042 | .002 | .964 | .001 | .957 | .028 | -.001 |
| 3 | Strong invariance | | 534.90 | 138 | ≤ .001 | .042 | .000 | .962 | .002 | .958 | .029 | -.001 |
| 4 | Strict invariance | | 544.97 | 153 | ≤ .001 | .039 | .003 | .963 | -.001 | .963 | .035 | -.006 |

## Table B3.

Measurement invariance of the factor model describing the structure of cognitive activation

|  | | | χ² | *df* | *p* | *RMSEA* | *ΔRMSEA* | *CFI* | *ΔCFI* | *TLI* | *SRMR* | *ΔSRMR* |
| --- | --- | --- | --- | --- | --- | --- | --- | --- | --- | --- | --- | --- |
| *Difficult course* | | |  |  |  |  |  |  |  |  |  |  |
| 1 | Configural invariance | | 563.38 | 118 | ≤ .001 | .048 |  | .955 |  | .942 | .035 |  |
| 2a | Weak invariance | | 716.53 | 128 | ≤ .001 | .053 | -.005 | .941 | .014 | .930 | .061 | -.026 |
| 2b | Partial weak invariance | | 679.28 | 127 | ≤ .001 | .051 | -.003 | .945 | .010 | .933 | .053 | -.018 |
| 3a | Strong invariance | | 1022.05 | 137 | ≤ .001 | .062 | -.011 | .911 | .034 | .901 | .086 | -.033 |
| 3b | Partial string invariance | | 749.40 | 136 | ≤ .001 | .052 | -.001 | .939 | .006 | .931 | .063 | -.010 |
| 4 | Strict invariance | | 847.34 | 151 | ≤ .001 | .053 | -.001 | .930 | .009 | .929 | .055 | .008 |
| *Important course* | | |  |  |  |  |  |  |  |  |  |  |
| 1 | | Configural invariance | 458.70 | 118 | ≤ .001 | .042 |  | .965 |  | .954 | .031 |  |
| 2 | | Weak invariance | 484.39 | 128 | ≤ .001 | .041 | .001 | .963 | .002 | .956 | .034 | -.003 |
| 3 | | Strong invariance | 517.26 | 138 | ≤ .001 | .041 | .000 | .961 | .002 | .957 | .036 | -.002 |
| 4 | | Strict invariance | 516.47 | 153 | ≤ .001 | .038 | .003 | .963 | -.002 | .963 | .039 | -.003 |

## Table C1.

Reliability (Ω) of used scales for each measurement point

|  | Fall quarter | | Winter quarter | | Spring quarter | | |
| --- | --- | --- | --- | --- | --- | --- | --- |
|  | T1 | T2 | T1 | T2 | T1 | T2 |  |
| *Difficult course* |  |  |  |  |  |  |  |
| Classroom management | .88 | .89 | .91 | .89 | .92 | .92 |  |
| Cognitive activation | .82 | .88 | .84 | .89 | .86 | .92 |  |
| Student support | .86 | .89 | .89 | .89 | .89 | .90 |  |
| Expectancies for success | .87 | -- | .89 | -- | .91 | -- |  |
| Interest value | .76 | -- | .76 | -- | .82 | -- |  |
| *Important course* |  |  |  |  |  |  |  |
| Classroom management | .89 | .90 | .91 | .90 | .92 | .93 |  |
| Cognitive activation | .85 | .90 | .89 | .90 | .87 | .91 |  |
| Student support | .85 | .87 | .88 | .89 | .90 | .90 |  |
| Expectancies for success | .88 | -- | .89 | -- | .91 | -- |  |
| Interest value | .71 | -- | .81 | -- | .78 | -- |  |

## Table D1.

Correlation between classroom management. student support and cognitive activation in the difficult and important courses in the fall quarter

|  |  | 1 | 2 | 3 | 4 | 5 | 6 | 7 | 8 | 9 | 10 | 11 |
| --- | --- | --- | --- | --- | --- | --- | --- | --- | --- | --- | --- | --- |
| 1 | CM_d1 | 1 |  |  |  |  |  |  |  |  |  |  |
| 2 | CM_d2 | .66 | 1 |  |  |  |  |  |  |  |  |  |
| 3 | CA_d1 | .79 | .55 | 1 |  |  |  |  |  |  |  |  |
| 4 | CA_d2 | .51 | .80 | .60 | 1 |  |  |  |  |  |  |  |
| 5 | SS_d1 | .83 | .59 | .81 | .56 | 1 |  |  |  |  |  |  |
| 6 | SS_d2 | .56 | .86 | .56 | .82 | .66 | 1 |  |  |  |  |  |
| 7 | CM_i1 | .39 | .27 | .34 | .20 | .32 | .24 | 1 |  |  |  |  |
| 8 | CM_i2 | .28 | .34 | .24 | .26 | .21 | .25 | .59 | 1 |  |  |  |
| 9 | CA_i1 | .35 | .24 | .39 | .27 | .32 | .22 | .78 | .50 | 1 |  |  |
| 10 | CA_i2 | .23 | .27 | .25 | .33 | .22 | .23 | .48 | .77 | .60 | 1 |  |
| 11 | SS_i1 | .37 | .23 | .34 | .22 | .34 | .24 | .86 | .55 | .80 | .52 | 1 |
| 12 | SS_i2 | .26 | .30 | .22 | .27 | .22 | .30 | .53 | .85 | .53 | .77 | .62 |

*Notes.* CM = classroom management, CA = cognitive activation, SS = student support, d = difficult course, I = important course, 1 = first measurement point in the quarter, 2 = second measurement point in the quarter, all estimates are significant with *p* ≤ .05.

## Table D2.

Correlation between classroom management. student support and cognitive activation in the difficult and important courses in the winter quarter

|  |  | 1 | 2 | 3 | 4 | 5 | 6 | 7 | 8 | 9 | 10 | 11 |
| --- | --- | --- | --- | --- | --- | --- | --- | --- | --- | --- | --- | --- |
| 1 | CM_d1 | 1 |  |  |  |  |  |  |  |  |  |  |
| 2 | CM_d2 | .64 | 1 |  |  |  |  |  |  |  |  |  |
| 3 | CA_d1 | .82 | .58 | 1 |  |  |  |  |  |  |  |  |
| 4 | CA_d2 | .52 | .79 | .66 | 1 |  |  |  |  |  |  |  |
| 5 | SS_d1 | .87 | .56 | .84 | .54 | 1 |  |  |  |  |  |  |
| 6 | SS_d2 | .63 | .83 | .65 | .80 | .68 | 1 |  |  |  |  |  |
| 7 | CM_i1 | .43 | .40 | .40 | .30 | .37 | .36 | 1 |  |  |  |  |
| 8 | CM_i2 | .30 | .36 | .30 | .29 | .26 | .32 | .55 | 1 |  |  |  |
| 9 | CA_i1 | .37 | .32 | .42 | .32 | .33 | .32 | .81 | .49 | 1 |  |  |
| 10 | CA_i2 | .24 | .27 | .29 | .30 | .22 | .27 | .48 | .82 | .58 | 1 |  |
| 11 | SS_i1 | .40 | .32 | .38 | .30 | .40 | .34 | .85 | .51 | .85 | .51 | 1 |
| 12 | SS_i2 | .25 | .31 | .28 | .29 | .27 | .33 | .50 | .84 | .52 | .82 | .58 |

*Notes.* CM = classroom management, CA = cognitive activation, SS = student support, d = difficult course, I = important course, 1 = first measurement point in the quarter, 2 = second measurement point in the quarter, all estimates are significant with *p* ≤ .05.

## Table D3.

Correlation between classroom management. student support and cognitive activation in the difficult and important courses in the winter quarter

|  |  | 1 | 2 | 3 | 4 | 5 | 6 | 7 | 8 | 9 | 10 | 11 |
| --- | --- | --- | --- | --- | --- | --- | --- | --- | --- | --- | --- | --- |
| 1 | CM_d1 | 1 |  |  |  |  |  |  |  |  |  |  |
| 2 | CM_d2 | .51 | 1 |  |  |  |  |  |  |  |  |  |
| 3 | CA_d1 | .83 | .45 | 1 |  |  |  |  |  |  |  |  |
| 4 | CA_d2 | .43 | .82 | .53 | 1 |  |  |  |  |  |  |  |
| 5 | SS_d1 | .87 | .44 | .83 | .45 | 1 |  |  |  |  |  |  |
| 6 | SS_d2 | .45 | .84 | .46 | .83 | .51 | 1 |  |  |  |  |  |
| 7 | CM_i1 | .45 | .27 | .44 | .23 | .39 | .21 | 1 |  |  |  |  |
| 8 | CM_i2 | .22 | .40 | .17 | .28 | .17 | .28 | .47 | 1 |  |  |  |
| 9 | CA_i1 | .40 | .25 | .47 | .27 | .36 | .21 | .82 | .39 | 1 |  |  |
| 10 | CA_i2 | .24 | .39 | .25 | .38 | .21 | .33 | .41 | .82 | .47 | 1 |  |
| 11 | SS_i1 | .40 | .23 | .40 | .19 | .40 | .22 | .87 | .47 | .81 | .42 | 1 |
| 12 | SS_i2 | .25 | .34 | .23 | .28 | .24 | .30 | .46 | .89 | .45 | .84 | .52 |

*Notes.* CM = classroom management, CA = cognitive activation, SS = student support, d = difficult course, I = important course, 1 = first measurement point in the quarter, 2 = second measurement point in the quarter, all estimates are significant with *p* ≤ .05.

## Table E1.

Model Fit Indices of the STMS Model (classroom management) for models with motivational beliefs included

|  | χ² | *df* | *p* | *RMSEA* | *CFI* | *TLI* | *SRMR* | |
| --- | --- | --- | --- | --- | --- | --- | --- | --- |
| *Model A: Stable context, same course, variable time* | | | | | | | |  |
| DIFFICULT: FALL^1^ | 200.40 | 47 | ≤ .001 | .044 | .979 | .975 | .051 | |
| DIFFICULT: WINTER^1^ | 200.75 | 47 | ≤ .001 | .048 | .974 | .970 | .037 | |
| DIFFICULT: SPRING^1^ | 207.38 | 47 | ≤ .001 | .050 | .977 | .973 | .037 | |
| IMPORTANT: FALL^1^ | 196.69 | 47 | ≤ .001 | .044 | .976 | .972 | .084 | |
| IMPORTANT: WINTER^1^ | 117.30 | 47 | ≤ .001 | .033 | .988 | .986 | .039 | |
| IMPORTANT: SPRING^1^ | 101.80 | 47 | ≤ .001 | .032 | .990 | .988 | .050 | |
| *Model B: Stable context, different course, variable time* | | | | | | | |  |
| DIFFICULT: FALL - SPRING^2^ | 1,998.43 | 528 | ≤ .001 | .040 | .935 | .935 | .074 | |
| IMPORTANT: FALL - SPRING^2^ | 1,555.69 | 528 | ≤ .001 | .033 | .954 | .954 | .074 | |
| *Model C: Variable context – stable time* | | | | | | | |  |
| FALL-W2^3^ | 634.21 | 103 | ≤ .001 | .056 | .946 | .938 | .080 | |
| FALL-W7^3^ | 537.91 | 103 | ≤ .001 | .050 | .956 | .949 | .058 | |
| WINTER -W2^3^ | 544.74 | 103 | ≤ .001 | .056 | .956 | .948 | .066 | |
| WINTER -W7^3^ | 570.96 | 103 | ≤ .001 | .058 | .948 | .940 | .069 | |
| SPRING -W2^3^ | 373.73 | 103 | ≤ .001 | .047 | .969 | .964 | .063 | |
| SPRING -W7^3^ | 428.43 | 103 | ≤ .001 | .051 | .964 | .958 | .063 | |
| *Model D* | 5,173.18 | 2,127 | ≤ .001 | .029 | .938 | .938 | .061 | |

*Notes*. ^1^ Same course (type = complex), ^2^ Different courses (but same context: different or important course) ^3^  two courses (different course: important and difficult). MB = motivational beliefs

## Table E2.

Model Fit Indices of the STMS Model (Student Support) for models with motivational beliefs included

|  | χ² | *df* | *p* | *RMSEA* | *CFI* | *TLI* | *SRMR* |
| --- | --- | --- | --- | --- | --- | --- | --- |
| *Model A: Stable context, same course, variable time* | | | | | | | |
| DIFFICULT: FALL^1^ | 339.06 | 47 | ≤ .001 | .06 | .96 | .96 | .04 |
| DIFFICULT: WINTER^1^ | 301.75 | 47 | ≤ .001 | .06 | .96 | .96 | .04 |
| DIFFICULT: SPRING^1^ | 211.59 | 47 | ≤ .001 | .05 | .98 | .97 | .04 |
| IMPORTANT: FALL^1^ | 312.75 | 47 | ≤ .001 | .06 | .96 | .95 | .08 |
| IMPORTANT: WINTER^1^ | 277.17 | 47 | ≤ .001 | .06 | .97 | .96 | .04 |
| IMPORTANT: SPRING^1^ | 161.65 | 47 | ≤ .001 | .05 | .98 | .98 | .03 |
| *Model B: Stable context, different course, variable time* | | | | | | | |
| DIFFICULT: FALL - SPRING^2^ | 2,384.53 | 526 | ≤ .001 | .05 | .92 | .92 | .07 |
| IMPORTANT: FALL - SPRING^2^ | 2,015.33 | 526 | ≤ .001 | .04 | .93 | .93 | .07 |
| *Model C: Variable context – stable time* | | | | | | |  |
| FALL-W2^3^ | 783.54 | 102 | ≤ .001 | .06 | .93 | .92 | .09 |
| FALL-W7^3^ | 560.30 | 102 | ≤ .001 | .05 | .95 | .95 | .06 |
| WINTER -W2^3^ | 636.16 | 102 | ≤ .001 | .06 | .94 | .93 | .06 |
| WINTER -W7^3^ | 631.37 | 102 | ≤ .001 | .06 | .95 | .94 | .06 |
| SPRING -W2^3^ | 446.51 | 102 | ≤ .001 | .05 | .96 | .95 | .06 |
| SPRING -W7^3^ | 488.97 | 102 | ≤ .001 | .06 | .96 | .95 | .07 |
| *Model D* | 6,758.70 | 2,127 | ≤ .001 | .04 | .91 | .90 | .06 |

*Notes*. ^1^ Same course (type = complex), ^2^ Different courses (but same context: different or important course) ^3^  two courses (different course: important and difficult)

## Table E3.

Model Fit Indices of the STMS Model (Cognitive Activation) for models with motivational beliefs included

|  | χ² | *df* | *p* | *RMSEA* | *CFI* | *TLI* | *SRMR* |
| --- | --- | --- | --- | --- | --- | --- | --- |
| *Model A: Stable context, same course, variable time* | | | | | | | |
| DIFFICULT: FALL^1^ | 252.55 | 47 | ≤ .001 | .05 | .97 | .97 | .05 |
| DIFFICULT: WINTER^1^ | 262.47 | 47 | ≤ .001 | .06 | .96 | .96 | .06 |
| DIFFICULT: SPRING^1^ | 273.68 | 47 | ≤ .001 | .06 | .96 | .96 | .06 |
| IMPORTANT: FALL^1^ | 265.09 | 47 | ≤ .001 | .05 | .96 | .96 | .06 |
| IMPORTANT: WINTER^1^ | 223.34 | 47 | ≤ .001 | .05 | .97 | .97 | .04 |
| IMPORTANT: SPRING^1^ | 163.21 | 47 | ≤ .001 | .05 | .98 | .97 | .04 |
| *Model B: Stable context, different course, variable time* | | | | | | | |
| DIFFICULT: FALL - SPRING^2^ | 2,394.34 | 525 | ≤ .001 | .05 | .91 | .91 | .07 |
| IMPORTANT: FALL - SPRING^2^ | 1,926.45 | 527 | ≤ .001 | .04 | .93 | .93 | .07 |
| *Model C: Variable context – stable time* | | | | | | | |
| FALL-W2^3^ | 743.29 | 103 | ≤ .001 | .06 | .93 | .92 | .08 |
| FALL-W7^3^ | 637.06 | 103 | ≤ .001 | .06 | .95 | .94 | .08 |
| WINTER -W2^3^ | 803.30 | 103 | ≤ .001 | .07 | .92 | .91 | .06 |
| WINTER -W7^3^ | 637.41 | 103 | ≤ .001 | .06 | .94 | .93 | .08 |
| SPRING -W2^3^ | 568.95 | 103 | ≤ .001 | .06 | .94 | .93 | .07 |
| SPRING -W7^3^ | 498.83 | 103 | ≤ .001 | .06 | .96 | .95 | .07 |
| *Model D* | 6,728.69 | 2,126 | ≤ .001 | .04 | .90 | .90 | .06 |

*Notes*. ^1^ Same course (type = complex), ^2^ Different courses (but same context: different or important course) ^3^  two courses (different course: important and difficult)

## Table F1.

*Covariances (*φ*) and standard errors (S.E.) between perceived instructional quality (pINQ) with interest value beliefs (IV) and expectancy for success (EXP) in Model A to C*

|  | *Classroom Management* | | | | *Student Support* | | | *Cognitive Activation* | | | |
| --- | --- | --- | --- | --- | --- | --- | --- | --- | --- | --- | --- |
|  | A1^a^  pINQ–EXP  φ *(S.E.)* | A2 ^a^  pINQ – IV  φ *(S.E.)* | A3 ^a^  IV – EXP  φ *(S.E.)* | A1 ^a^  pINQ–EXP  φ *(S.E.)* | | A2 ^a^  pINQ – IV  φ *(S.E.)* | A3 ^a^  IV – EXP  φ *(S.E.)* | A1 ^a^  pINQ–EXP  φ *(S.E.)* | A2 ^a^  pINQ – IV  φ *(S.E.)* | A3 ^a^  IV – EXP  φ *(S.E.)* |  |
| *Model A: Stable context, same course, variable time* | | | | | | | | | | |  |
| DIFFICULT: FALL^1^ | .42 (.05) | .65 (.07) | .75 (.06) | .50 (.05) | | .69 (.07) | .75 (.06) | .50 (.05) | .85 (.07) | .75 (.06) |  |
| DIFFICULT: WINTER^1^ | .36 (.05) | .78 (.07) | .88 (.06) | .44 (.05) | | .82 (.07) | .89 (.06) | .44 (.05) | 1.00 (.07) | .89 (.06) |  |
| DIFFICULT: SPRING^1^ | .36 (.06) | .78 (.09) | .88 (.09) | .56 (.08) | | 1.00 (.10) | 1.08 (.10) | .65 (.06) | 1.17 (.09) | 1.08 (.08) |  |
| IMPORTANT: FALL^1^ | .33 (.04) | .50 (.05) | .54 (.04) | .35 (.03) | | .49 (.06) | .54 (.04) | .34 (.03) | .60 (.05) | .54 (.04) |  |
| IMPORTANT: WINTER^1^ | .35 (.04) | .59 (.06) | .65 (.05) | .38 (.04) | | .69 (.07) | .65 (.06) | .36 (.05) | .81 (.07) | .65 (.06) |  |
| IMPORTANT: SPRING^1^ | .38 (.05) | .60 (.06) | .75 (.08) | .38 (.06) | | .62 (.07) | .75 (.08) | .40 (.06) | .75 (.07) | .75 (.08) |  |
| *Model B: Stable context, different course, variable time* | | | | | | | | | | |  |
| DIFFICULT: FALL - SPRING^2^ | .30 (.03) | .48 (.04) | .44 (.05) | .36 (.03) | | .51 (.04) | .43 (.05) | .39 (.03) | .60 (.04) | .34 (.04) |  |
| IMPORTANT: FALL - SPRING^2^ | .25 (.02) | .38 (.03) | .28 (.03) | .27 (.02) | | .41 (.03) | .33 (.04) | .29 (.03) | .48 (.03) | .33 (.04) |  |
| *Model C: Variable context, different courses, and stable time* | | | | | | | | | | |  |
| FALL-W2^3^ | .28 (.04) | .45 (.05) | .30 (.05) | .36 (.04) | | .52 (.04) | .36 (.06) | .33 (.05) | .41 (.05) | .30 (.04) |  |
| FALL-W7^3^ | .28 (.04) | .51 (.05) | .29 (.05) | .34 (.04) | | .53 (.05) | .36 (.05) | .35 (.04) | .64 (.05) | .35 (.05) |  |
| WINTER -W2^3^ | .31 (.04) | .67 (.06) | .34 (.06) | .35 (.05) | | .66 (.06) | .34 (.06) | .38 (.05) | .82 (.06) | .34 (.05) |  |
| WINTER -W7^3^ | .24 (.04) | .53 (.06) | .27 (.05) | .28 (.05) | | .56 (.06) | .30 (.05) | .24 (.05) | .62 (.06) | .29 (.05) |  |
| SPRING -W2^3^ | .38 (.05) | .72 (.07) | .47 (.09) | .38 (.06) | | .71 (.07) | .63 (.10) | .42 (.05) | .84 (.08) | .60 (.09) |  |
| SPRING -W7^3^ | .41 (.05) | .85 (.08) | .55 (.07) | .46 (.06) | | .92 (.08) | .61 (.08) | .51 (.06) | .98 (.08) | .58 (.08) |  |

*Notes*. ^a^ The tested paths A1, A2, A3 are visualized in Figures 3 and 4, all estimates are significant with *p* ≤ .001.

## Table F2

*Covariances (*φ*) and standard errors (S.E.) between perceived instructional quality (pINQ) with interest value beliefs (IV) and expectancy for success (EXP) in Model D*

|  |  |  |  | *Classroom Management* | *Student Support* | *Cognitive Activation* |
| --- | --- | --- | --- | --- | --- | --- |
|  |  |  |  | φ (S.E.) | φ (S.E.) | φ (S.E.) |
| A1 | pINQ.IMP | with | pINQ.DIFF | .53 (.03) | .51 (.03) | .54 (.04) |
| A2 | EXP.IMP | with | EXP.DIFF | .34 (.03) | .32 (.03) | .32 (.03) |
| A3 | IV.IMP | with | IV.DIFF | .54 (.05) | .51 (.04) | .53 (.04) |
| B1 | pINQ.IMP | with | EXP.IMP | .26 (.02) | .28 (.02) | .28 (.02) |
| B2 | pINQ.IMP | with | EXP.DIFF | .16 (.02) | .19 (.03) | .20 (.03) |
| B3 | pINQ.IMP | with | IV.IMP | .39 (.03) | .40 (.03) | .48 (.03) |
| B4 | pINQ.IMP | with | IV.DIFF | .34 (.03) | .36 (.03) | .42 (.03) |
| C1 | pINQ.DIFF | with | EXP.DIFF | .32 (.03) | .34 (.03) | .38 (.03) |
| C2 | pINQ.DIFF | with | EXP.IMP | .24 (.02) | .52 (.02) | .25 (.03) |
| C3 | pINQ.DIFF | with | IV.DIFF | .51 (.04) | .49 (.04) | .61 (.04) |
| C4 | pINQ.DIFF | with | IV.IMP | .32 (.03) | .30 (.03) | .35 (.03) |

*Notes*. pINQ = perceived instructional quality (either classroom management, student support or cognitive activation), T1 = time 1 in each quarter, T2 = time 2 in each quarter, Q1 = first quarter of academic year (fall), Q2 = second quarter of academic year (winter), Q3 = third quarter of academic year (spring), DIFF = difficult course, IMP = important courses. The paths a1 to c4 are visualized in Figure 4. All estimates are significant with *p* ≤ .05.

## Table G1.

Overview of items on used items

| **Student Support** | |
| --- | --- |
| SS1 | To what extent can you ask your instructor for help if you do not understand course-related material in your most *difficult/important course*? |
| SS2 | To what extent is the instructor helpful in guiding the class towards understanding course topics in a way that helps you clarify your thinking in your most *difficult/important course*? |
| SS3 | To what extent does the instructor provide feedback that helps you understand your strengths and weaknesses relative to the goals and objectives of your most *difficult/important course*? |
| **Classroom Management** | |
| CM1 | To what extent does the instructor clearly communicate important course goals in your most *difficult/important course*? |
| CM2 | To what extent does the instructor provide clear instructions on how to participate in learning activities in your most *difficult/important course*? |
| CM3 | To what extent does the instructor clearly communicate important due dates and time frames for learning activities in your most *difficult/important course*? |
| **Cognitive Activation** | |
| CA1 | To what extent does the instructor encourage the class to explore new concepts in your most difficult/important course? |
| CA2 | To what extent does the instructor help to focus the discussion on relevant issues in a way that helps you to learn the content in your most *difficult/important course*? |
| CA3 | To what extent does the instructor in your most *difficult/important course* help to activate prior knowledge and connect it with new course content? |
| **Expectancies for success** | |
| EXP1 | How good do you think you will be at learning the new material in your most *difficult/important course*? |
| EXP2 | Compared to other courses. how good are you at learning things in your most *difficult/important course*? |
| EXP3 | Compared to your peers in this course. how good are you at learning things in your most *difficult/important course*? |
| **Interest value beliefs**  On a scale from 1-7. how much do you expect that your most *difficult/important course* will … | |
| IV1 | … be interesting to you? |
| IV2 | … be intellectually challenging in a positive way? |

## 
